# MiR-93-5p promotes granulosa cell apoptosis and ferroptosis by the NF-kB signaling pathway in polycystic ovary syndrome

**DOI:** 10.3389/fimmu.2022.967151

**Published:** 2022-10-19

**Authors:** Wei Tan, Fangfang Dai, Dongyong Yang, Zhimin Deng, Ran Gu, Xiaomiao Zhao, Yanxiang Cheng

**Affiliations:** ^1^ Department of Obstetrics and Gynecology, Renmin Hospital of Wuhan University, Wuhan, China; ^2^ Department of Reproductive Medicine, Guangdong Provincial People’s Hospital, Guangdong Academy of Medical Sciences, Guangzhou, China

**Keywords:** polycystic ovary syndrome, miR-93-5p, ferroptosis, NF-κB, apoptosis

## Abstract

Polycystic ovary syndrome (PCOS) is one of the most common endocrine disorders in women of reproductive age. miR-93-5p has been reported to be elevated in granulosa cells of PCOS patients. However, the mechanism by which miR-93-5p drives granulosa cell (GC) progression remains unclear. Thus, this study focuses on the roles and mechanisms of miR-93-5p in the GCs of PCOS.

**Methods:**

KGN cells have similar ovarian physiological characteristics and are used to study the function and regulatory mechanism of GCs. In this study, KGN cells were transfected with si-NC, si-miR93-5p, oe-NC and oe-miR93-5p. A cell counting kit-8 assay, flow cytometry and western blotting were performed to observe the proliferation and apoptosis of KGN in different groups. Subsequently, the levels of reactive oxygen species, malondialdehyde, GPX4, SLC7A11 and Nrf2, which are indicators of ferroptosis, were measured by a dihydroethidium fluorescent dye probe, biochemical kit, western blotting and reverse transcription quantitative polymerase chain reaction. Ultimately, bioinformatic analysis and experimental methods were used to examine the interaction between miR-93-5p and the NF-κB signaling pathway.

**Results:**

miR-93-5p was upregulated in the GCs of PCOS patients. Overexpression of miR-93-5p promoted apoptosis and ferroptosis in KGN cells, while knockdown of miR-93-5p showed the reverse effect. Biological analysis and subsequent experiments demonstrated that miR-93-5p negatively regulates the NF- κB signaling pathway.

**Conclusion:**

miR-93-5p promotes the apoptosis and ferroptosis in GC by regulating the NF-κB signaling pathway. Silencing of miR-93-5p protects against GC dysfunction. Our study identified miR-93-5p as a new molecular target for improving the function of GCs in PCOS patients.

## Introduction

Polycystic ovary syndrome (PCOS) is characterized by oligo-ovulation or anovulation, high androgen or insulin resistance, and polycystic ovaries, and it represent the most common endocrine aberration in women of reproductive age (1). According to the latest international evidence-based guidelines, the diagnosis of PCOS is based on irregular menses, clinical or biochemical hyperandrogenism and ultrasound criteria (2). Furthermore, the pathogenesis of PCOS is intricate and multifactorial, including genetic, epigenetic, environmental and lifestyle factors (3). To date, the precise pathophysiology remains to be elucidated.

MicroRNAs are endogenous non-coding RNAs at ∼22 nt in length that regulate a variety of biological activities, such as metabolic homeostasis, cell differentiation, oxidative stress, and apoptosis (4, 5). These RNAs play important regulatory roles in gene expression by binding to the 3’UTR of target mRNAs for cleavage or translational repression (6). Accumulating studies have revealed that aberrant expression of miR-93-5p contributes to the biological processes of various types of disease in women. For example, it regulates trophoblast cell proliferation, migration, invasion, and apoptosis in recurrent spontaneous abortion, suppresses tumorigenesis and enhances the chemosensitivity of breast cancer (7–9). Furthermore, studies have reported that miR-93-5p expression levels are significantly up-regulated in PCOS patients and can regulate granulosa cell (GC) proliferation and insulin resistance in adipose tissue (10–14).

Cells are the basic unit of organisms, and their proliferation and death play a major role in the myriad manifestations of life (15). Compared with apoptosis, ferroptosis is a new iron-dependent form of non-apoptotic cell death, characterized by iron accumulation, fatty acid supply and lipid peroxidation (16). Notably, studies of ferroptosis in diverse diseases have increased exponentially in the last few years and inhibition or activation can benefit many diseases, such as neurodegeneration and tumors (16–19). However, few studies have focused on about the role of ferroptosis in PCOS.

GCs are the largest group of cells in the ovary, and they surround oocytes, and provide nutrients and maturation-enabling factors (20). These cells play a crucial role in follicle development and oocyte competence, and the morphology and number of encircling GCs have been used as biomarkers for developmental competency, embryo and pregnancy outcomes (21). Hence, the purpose of this study was to investigate the effect of miR-93-5p on ferroptosis of GCs in PCOS, and explore the possible mechanisms through bioinformatic analysis and *in vitro* experimental verification. Furthermore, we also conducted a parallel analysis of proliferation and apoptosis in GCs. Our results revealed that miR-93-5p was elevated in PCOS, and involved in proliferation, apoptosis and ferroptosis by regulating the nuclear factor-κB (NF-κB) signaling pathway. Our study is helpful for revealing the mechanism of PCOS and may provide new insights for the targeted therapy of PCOS.

## Materials and methods

### Data collection and procession

The miRNA expression profiles (GSE86241, GSE68285, and GSE138572) and mRNA expression profiles of PCOS GCs (GSE95728 and GSE34526) were downloaded from Gene Expression Omnibus (GEO, https://www.ncbi.nlm.nih.gov/gds). In order to explore the targets of miR-93-5p, we conducted biological prediction on miRNet (https://www.mirnet.ca) and Starbase (http://starbase.sysu.edu.cn/), and finally collected 5669 potential targets (22, 23). Additionally, 3220 PCOS-associated genes were obtained from Disgenet (http://www.disgenet.org/), Phenopedia (http://www.hugenavigator.net/HuGENavigator/startPagePhenoPedia.do) and GeneCards (www.genecards.org) website (24–26). Then, the overlapped genes of PCOS-associated genes and miR-93-5p targets was screened. Differentially expressed genes (DEGs) was screened by limma package and *p-value* < 0.05 was considered statistically significant (27). Only the DEGs in both GSE95728 and GSE34526 were retained for subsequent analysis.

### Functional analysis

The Gene ontology (GO) analysis and the Kyoto Encyclopedia of Genes and Genomes (KEGG) were applied to explore the potential roles and signaling pathway of the miR-93-5p-related and DEGs in PCOS using the clusterProfiler package (28). *p* < 0.05 was considered significant.

### Cell culture and transfection

The human GC tumor-derived cell line KGN was purchased from CELLCOOK biotechnology. Cells were cultured in Dulbecco’s modified Eagle’s medium (DMEM)/F-12 (Meilunbio, China) supplemented with 10% fetal bovine serum (Gibco, Life Technologies, Grand Island, NY) and 100 units/mL penicillin and streptomycin (Invitrogen, Waltham, MA, United States) at 37°C with 5 % CO_2_. Lentivirus was purchased from Shanghai GeneChem Co., LTD. Cells were treated with si-miR93-5p (silencing of miR-93-5p), si-NC, oe-miR93-5p (overexpression of miR-93-5p) lentivirus and oe-NC. A fluorescence microscope with a digital camera (Olympus, Tokyo, Japan) was used to observe the fluorescence intensity of the cells. After 48 h of infection, the cells were selected with 5mg/mL puromycin (Invitrogen, United States). For inhibitor experiment, NF-κB inhibitor (BAY 11-7082) was purchased from MedChemExpress (MCE, China) and KGN cells were treated with 5 μM BAY 11-7082 for 24 h. Ferroptosis inhibitor (Fer-1) and ferroptosis inducer (erastin) were purchased from Good Laboratory Practice Bioscience (GLPBio, China).

### Animals

C57BL/6 J (3-week-old) female mice, weighing 15–20 g, were purchased from Beijing Vitalriver Laboratory Animal Technology Co Ltd., China. Experimental animals were randomly divided into control (glycerol treatment) and experimental groups (PCOS, DHEA treatment). The modeling method was as described previously (29). The PCOS model and control was verified successfully as our previous results, and ovarian tissue was extracted for RT-qPCR detection (30).

### Reverse transcription quantitative polymerase chain reaction (RT-qPCR)

Total RNA was extracted from cells by TRIzol reagent (Invitrogen) according to the manufacturer’s instructions. Total RNA (1 μg) was reverse transcribed using a Reverse Transcription kit (Yeasen Biotechnology Co. Ltd.), and the resultant cDNA was utilized as the qPCR template. The reaction system of RT-qPCR was 2 μl, composed of 0.4 μl F, 0.4 μl R, 7.2 μl DEPC, 2 μl cDNA and 10 μl of SYBR. Then, RT-qPCR was carried out on a Light Cycler 480 system using SYBR GREEN PCR Master Mix (Yeasen Biotechnology Co. Ltd.) according to the real-time PCR manufacturer’s instructions. U6 and GAPDH served as internal controls for miR-93-5p and mRNA, respectively. The relative levels of genes were calculated using the 2^-△△Ct^ method. **Table 1** lists the primers that were utilized.

**Table 1:** Sequences of primers used for RT-qPCR.

	Forward primer 5’→3’	Reverse primer 5’→3’
Hsa-miR-93-5p mmu-miR-93-5p	GCAAAGTGCTGTTCGTGC AAGTGCTGTTCGTGCAGGT	AGTGCAGGGTCCGAGGTATT CTCGGGAAGTGTAGCTCA
U6 p53 Nrf2 SLC7A11 GPX4 GAPDH	AGCACATATACTAAAATTGGAACGAT GCTGGTTAGGAGGGAGTTG TCACAAGAGATGAACTTAGGGCAA TGTCTCCAGGTTATTCTATGTTG TTGCCGCCTACTGAAGC CCTTCCGTGTCCCCGAT	ACTGCAGGGTCCGAGGTATT GTGTGGGATGGGGTGAGATTTC AGCCACTTTATTCTTACCCCTC CCAGAGAAGAGCATTATCATTG ATGTGCCCGTCGATGTC GCCTGCTTCACCACCTTC

### Western blotting analysis

Total protein was extracted using RIPA buffer including PMSF protease inhibitors (Beyotime Biotechnology, China) and a phosphatase inhibitor (Servicebio, China) from cultured cells. Protein concentrations were measured by BCA protein assay kit (Beyotime Biotechnology, China). 10 µg of total protein lysates were electrophoresed in 10% SDS-polyacrylamide gels (Yeasen Biotechnology Co. Ltd.). After separated by SDS-PAGE gels, proteins were transferred onto 0.45 mm/0.22 mm polyvinylidene difluoride (Merck Millipore, Billerica, MA). The membranes were blocked for 2 h at room temperature with 5% non-fat milk. Remove blocking solution and add diluted primary antibody overnight at 4°C. Then, wash 5 times with TBST for 5 min each. Then, the membranes were incubated with primary antibodies against phospho-NF-κB p65 (1:1000; Affinity, China, Cat: #AF5006), phospho-IκBα-S32/S36 (1:1,000; ABclonal, China, Cat: #AP1201), IκBα (1:1,000; ABclonal, Cat: #A1167), TLR4 (1:5000; proteintech, China, Cat: 66350-1-lg), Bax (1:1,000; ABclonal, China, Cat: #A12009, Bcl-2 (1:1,000; ABclonal, China, Cat: #A0208), GPX4 (1:1,000; ABclonal, China, Cat: #A11241), Tubulin (1:1,000; ABclonal, China, Cat: #A15103), or GAPDH (1:50000, proteintech, China, Cat: 60004-1-lg) overnight at 4°C. Subsequently, the secondary antibodies were used (1:7500; ABclonal, China, Cat: #ASO14, #AS003) to incubate the membranes for 1 h at room temperature. The membranes were washed 3 times with TBST at room temperature for 5 minutes each time and the targeted proteins were detected by ECL reagents (ABclonal, China). The intensity of protein bands was quantified by Image J software (Ver 1.5.3)

### Cell viability and apoptosis assays

KGN cells were evenly plated in 96-well plates at 5000 cells/well. Cell proliferation ability was determined *via* cell counting kit-8 (CCK-8) assay (Glpbio Technology, China) at 0, 24, 48 and 72 h according to the manufacturer’s instructions. For the apoptosis analysis, an Annexin V-PE/7-AAD Apoptosis Detection Kit (Yeasen, Shanghai, China) was used to stain the cells. The adherent KGN cells were digested with trypsin without EDTA and centrifuged at 300 × g for 5 min at 4°C. Next, the cells were washed twice with pre-cooled PBS, 300g each time, and they were then centrifuged at 4°C for 5 min. The cells were resuspended with 250 mL binding buffer, and 100 mL of cell suspension was removed. Annexin V/PE (5 mL) and 7-AAD (10 mL) were added to the cell suspension. The mixture was mixed gently, and the reaction was performed at room temperature for 15 min in the dark. Then, the apoptotic cells were measured using a flow cytometer (Beckman Coulter, CA, USA). The percentage of apoptotic cells was analyzed with CytExpert software.

### Measurement of reactive oxygen species and malondialdehyde

Intracellular ROS production was measured by the dihydroethidium (DHE) fluorescent dye probe (Yeasen, Shanghai, China). The adherent KGN cells were digested with trypsin and centrifuged at 300 × g for 5 min at 4°C. Then, the cell suspension and staining solution were mixed in a 1:1 ratio and incubated for 60 min at 37°C. The MDA (BC0025, Beijing Solarbio Science and Technology Co. Ltd, China) was measured following the manufacturer’s instructions.

### Statistical analysis

GraphPad Prism 8 and R (4.1.2) were applied to analyze all experimental data. if the samples in two groups met the parameter conditions (normal distribution and homogeneity of variance), then a t test was employed; otherwise, the nonparametric two-sided Wilcoxon-rank sum test was performed. For multiple groups, ANOVA was performed if the samples met the parameter conditions; otherwise, Kruskal-Wallis tests were employed. A value of *p* < 0.05 indicated statistical significance.

## Results

### Expression of miR-93-5p in GCs of PCOS

**Figure 1:**
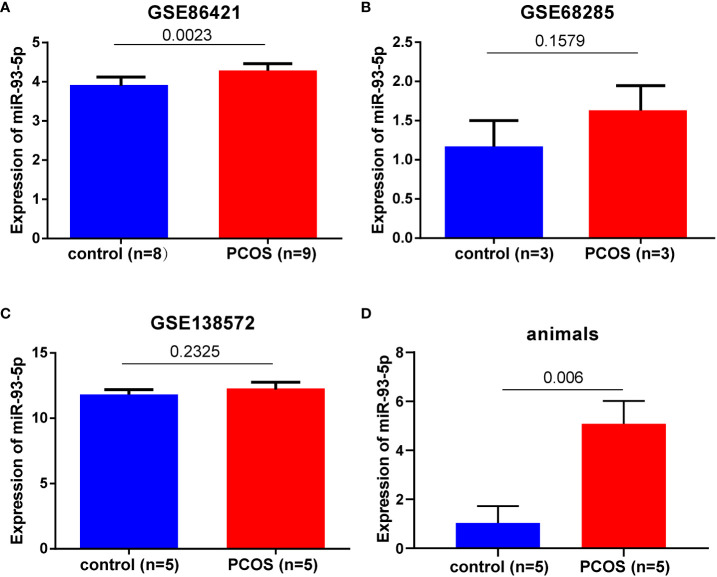
The expression level of miR-93-5p in GCs of PCOS and heath controls. miR-93-5p expression in **(A)** GSE86421, **(B)** GSE68285, **(C)** GSE138572, **(D)**ovarian tissues of PCOS mice (n=5) and controls (n=5).

In this study, three GEO datasets (GSE86241, GSE68285 and GSE138572) were collected. The expression of miR-93-5p in PCOS was elevated in GSE86241 (*p* < 0.05, **Figure 1A**). However, significant difference of miR-93-5p were not observed in GSE68285, and GSE138572 datasets, perhaps due to insufficient samples (**Figures 1B, C**). Additionally, we used 5 PCOS and control mice that were successfully modeled before extracting RNA from ovarian tissue and detecting the expression of miR-93-5p (**Figure 1D**) (30). The results validated that the level of miR-93-5p was increased in PCOS mice. Next, we retrieved miR-93-5p expression in GCs of PCOS based on published literature data (11, 14, 31). As shown in **Table 2**, the expression of miR-93-5p was higher in PCOS.

**Table 2:** The studies of miR-93-5p in granulosa cell from literature.

Author	Year	Country	PCOS cases	Normal cases	Results
Jiang et al (14)	2015	China	16	8	Up-regulation
Naji et al (11)	2017	Iran	41	25	Up-regulation
Sathypalan et al (31)	2015	UK	25	25	Up-regulation

### Overexpression of miR-93-5p promotes the apoptosis and ferroptosis in GCs

**Figure 2:**
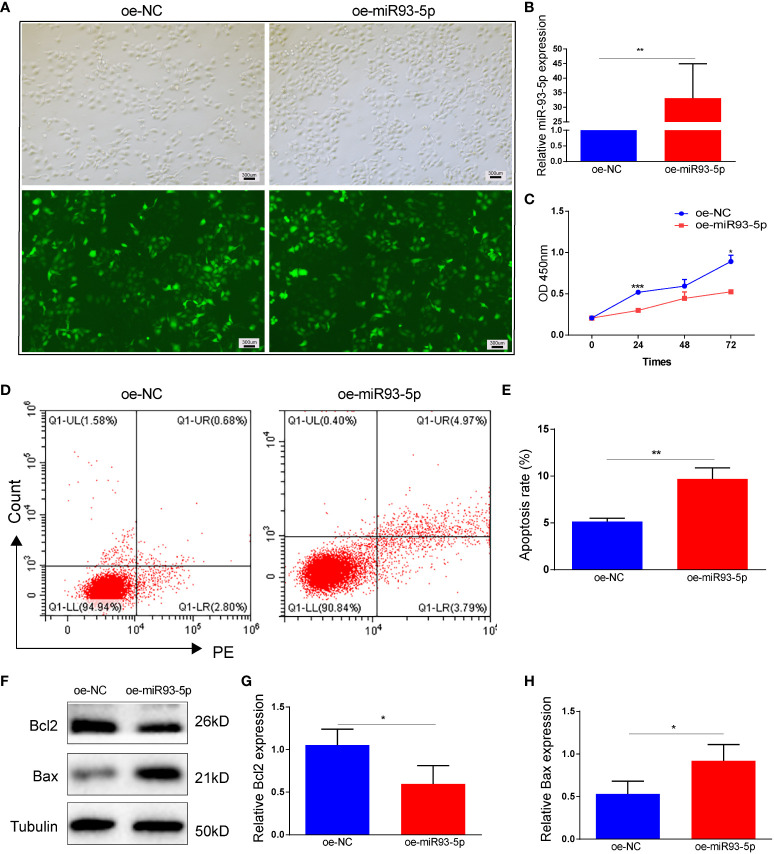
The effect of overexpression of miR-93-5p on the apoptosis in GC. **(A)** Cells were transfected with the oe-NC and oe-miR93-5p lentivirus for 72h. **(B)** The expression of miR-93-5p in GCs detected by RT-qPCR. **(C)** Cell viability in the oe-NC and oe-miR93-5p groups detected by CCK-8 assay. **(D, E)** Flow cytometry assay displayed the apoptosis level in two groups and bar charts are used for visualization. **(F–G)** Bax and Bcl-2 protein expression were determined by western blotting in two groups. *p* < 0.05 (“*”), *p* < 0.01 (“**”), *p* < 0.001 (“***”).

To investigate the role of miR-93-5p *in vitro*, we infected KGN cells with lentiviruses-overexpressing miR-93-5p gene (**Figure 2A**). RT-qPCR was used to verify the transfection efficiency of miR-93-5p, and the results showed that the level of miR-93-5p was 32.7-fold greater than that of the control (*p* < 0.01, **Figure 2B**). CCK8 was applied to detect the cell proliferation rate, and it revealed that elevated miR-93-5p could inhibit KGN cell proliferation (**Figure 2C**). Furthermore, the upregulation of miR-93-5p also promoted cell apoptosis (**Figures 2D, E**). Additionally, we observed decreased expression of Bcl2 and increased expression of Bax in the oe-miR93-5p group (**Figures 2F–H**). Ferroptosis is characterized by the accumulation of lipid ROS and MDA and the suppression of glutathione peroxidase 4 (GPX4) and solute carrier family member 11 (SLC7A11). GPX4, an enzyme required for the clearance of lipid ROS, plays a vital role in the cellular antioxidant system (32). When GPX4 is inhibited, lipid peroxides are amplified and accumulate, thereby inducing ferroptosis (15). To investigate the regulatory effects of miR-93-5p on ferroptosis, we compared the ROS, MDA, and GPX4 levels in the two groups. The results showed that the ROS and MDA levels increased significantly in the oe-miR93-5p group, while the GPX4 levels were decreased (**Figure S1**).

**Figure 3:**
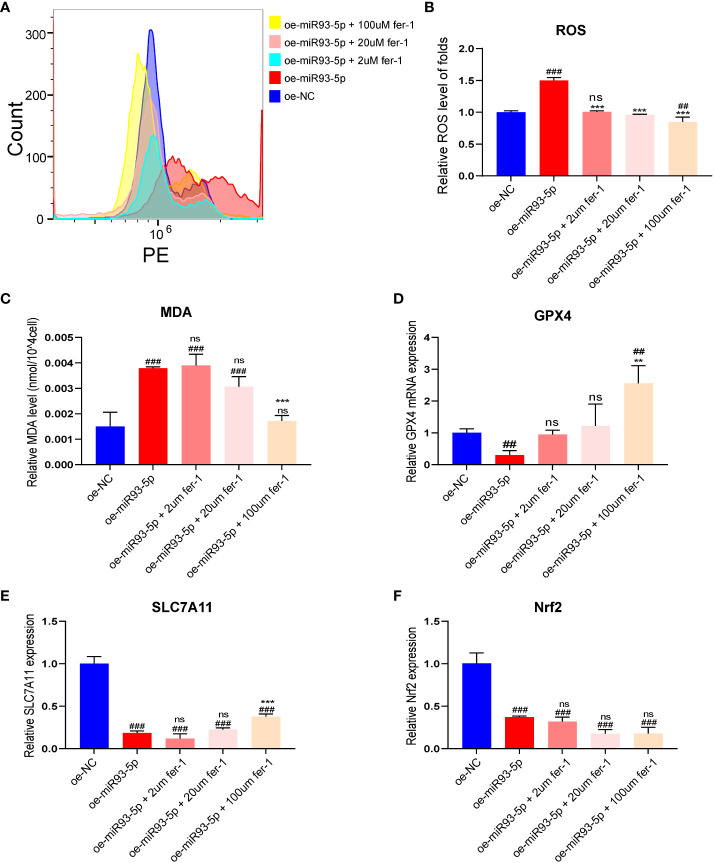
The effect of overexpression of miR-93-5p on the ferroptosis of GC. **(A, B)** The ROS level between the oe-NC, oe-miR93-5p and treated with fer-1 (2, 20, and 100 μM) groups. **(C)** MDA assay was used to detect lipid peroxidation in the presence of fer-1 (2, 20, and 100 μM) in the oe-miR93-5p group. GPX4 **(D)**, SLC7A11 **(E)** and Nrf2 **(F)** mRNA expression was assessed by qRT–PCR in the presence of fer-1 (2, 20, and 100 μM) in the oe-miR93-5p group. ^##^ *p* < 0.01 and ^###^ *p* < 0.001 compared with the oe-NC group. **p < 0.01, ***p < 0.001 compared with the oe-miR93-5p group. ns means no statistical difference. Fer-1, ferrostatin-1; GPX4, glutathione peroxidase 4; SLC7A11, solute carrier family member 11; MDA, malondialdehyde; ROS, reactive oxygen species.

To further validate the effect of miR-93-5p on ferroptosis, fer-1 (2, 20, 100 μM), a ferroptosis inhibitor, was applied in our study. Treatment of the oe-miR93-5p group with fer-1 significantly attenuated the ROS level (**Figures 3A, B**). A high concentration of fer-1 (100 μM) reversed the ROS, MDA and GPX4 alterations induced by miR-93-5p. However, treatment of the oe-miR93-5p group with fer-1 (2, 20 μM) did not alter the MDA and GPX4 levels compared with that of the oe-miR93-5p group (**Figures 3C, D**). SLC7A11 is the cystine/glutamate antiporter (also commonly known as xCT) that imports cystine for glutathione biosynthesis and antioxidant defense (33, 34). Badyley et al. reported that if the function of SLC7A11 is inhibited, then cysteine may break down, thereby inducing an impairment of the GPX4 antioxidant defense axis (33). We observed that the expression of SLC7A11 was repressed in the oe-miR93-5p group and treatment of the oe-miR93-5p group with fer-1 (100 μM) increased the SLC7A11 level compared with that of the oe-miR93-5p group (**Figure 3E**). Nuclear factor erythroid 2-related Factor 2 (NRF2) is the major transcription factor that regulates SLC7A11 and GPX4 transcription (34, 35). The level of Nrf2 was abated in the oe-miR93-5p group. Of note, treatment of the oe-miR93-5p group with fer-1 (2, 20 and 100 μM) did not influence the level of Nrf2 (**Figure 3F**).

### Inhibition of miR-93-5p inhibits the apoptosis and ferroptosis

**Figure 4:**
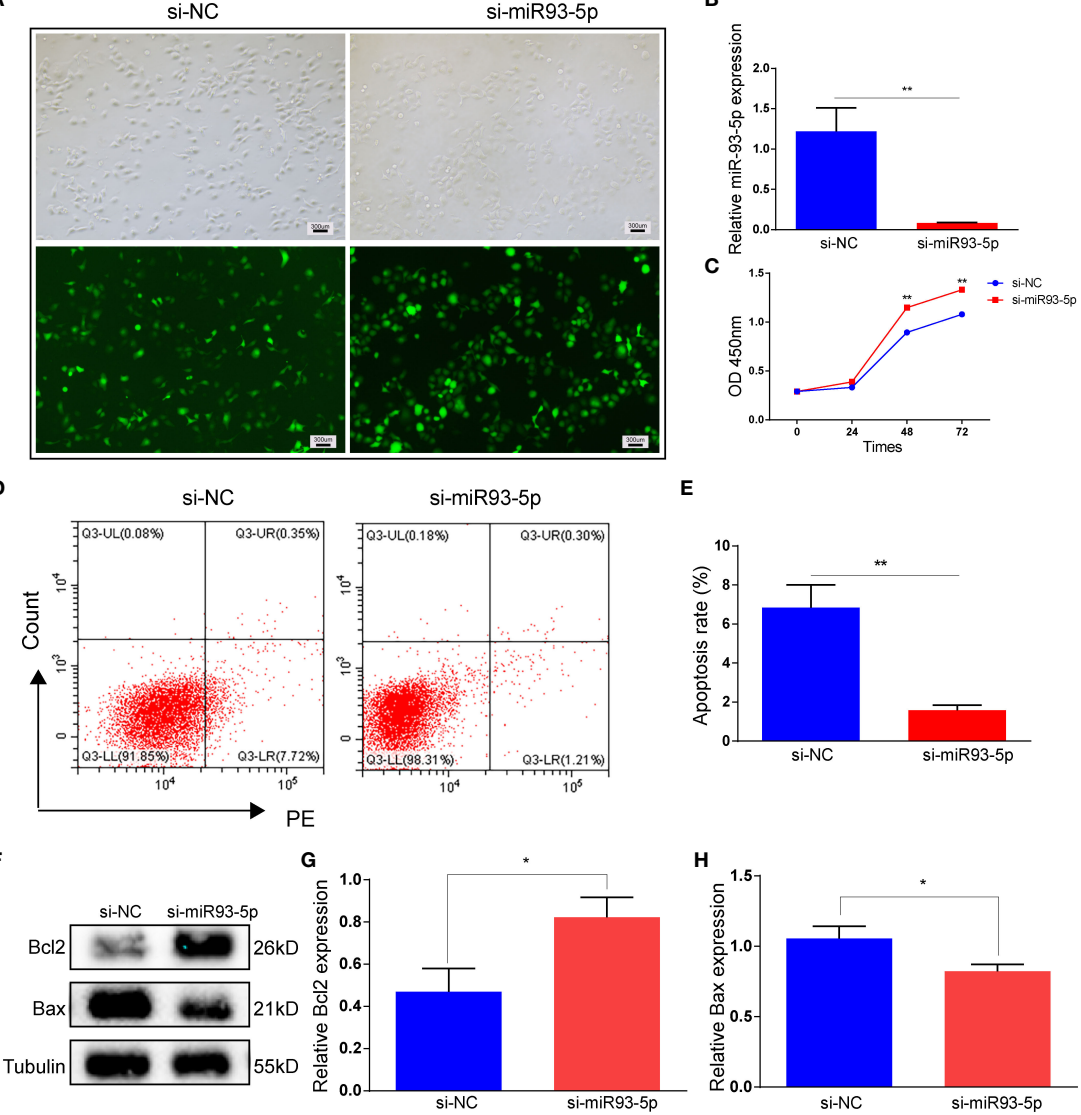
The effect of inhibition of miR-93-5p on the apoptosis in GC. **(A)** Cells were transfected with the si-NC and si-miR93-5p lentivirus. **(B)** The expression of miR-93-5p in GC detected by RT-qPCR. **(C)** Cell viability in the si-NC and si-miR93-5p groups detected by CCK-8 assay. **(D, E)** Flow cytometry assay displayed the apoptosis level in two groups and bar charts are used for visualization. **(F, G)** Bax and Bcl-2 protein expression were determined by western blotting in two groups. *p* < 0.05 (“*”), *p* < 0.01 (“**”). GC, granulosa cell; RT-qPCR, reverse transcription quantitative polymerase chain reaction; CCK-8, cell counting kit-8.

**Figure 5:**
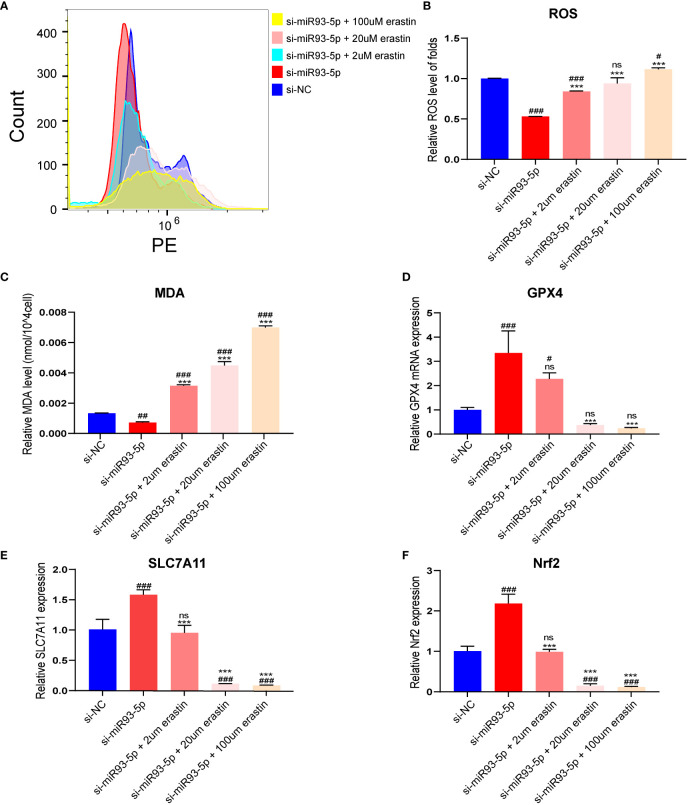
The effect of inhibition of miR-93-5p on the ferroptosis in GC. **(A, B)** The ROS level between the si-NC, si-miR93-5p and treated with erastin (2, 20, and 100 μM) groups. **(C)** MDA assay was used to detect lipid peroxidation in the presence of erastin (2, 20, and 100 μM) in the si-miR93-5p group. GPX4 **(D)**, SLC7A11 **(E)** and Nrf2 **(F)** mRNA expression was assessed by qRT–PCR in the presence of erastin (2, 20, and 100 μM) in the si-miR93-5p group. ^#^ *p* < 0.05, ^##^ *p* < 0.01 and ^###^ *p* < 0.001 compared with the si-NC group. ***p < 0.001 compared with the si-miR93-5p group. ns means no statistical difference. GPX4, glutathione peroxidase 4; SLC7A11, solute carrier family member 11; MDA, malondialdehyde; ROS, reactive oxygen species.

We verified the successful transfection of si-miR93-5p by RT-qPCR (**Figures 4A, B**). Compared with the oe-miR-93-5p group, the si-miR93-5p group showed downregulated cell proliferation and upregulated cell apoptosis (**Figures 4C–H**). In addition, the inhibition of miR-93-5p promoted ferroptosis (**Figure S2**). Erastin (2, 20 and 100 μM), a ferroptosis inducer, was applied in our study. Compared with the si-miR93-5p group, ROS and MDA were significantly elevated in the si-miR93-5p group treated with erastin (2, 20 and 100 μM), and the expression of GPX4, SLC7A11 and Nrf2 was reduced (**Figure 5**).

### Functional enrichment analysis of miR-93-5p-related genes

A total of 5669 potential miR-93-5p targets were collected based on the miRNet and StarBase websites and 3220 PCOS-associated genes were obtained from the Disgenet, Phenopedia and GeneCards websites. The intersection of PCOS related genes and miR-93-5p targets was calculated, and 1073 overlapping genes were finally identified (**Figure S3A**). The expression of 1073 overlapping genes in PCOS and normal GCs were studied using GEO datasets (GSE95728 and GSE34526). Then, we identified 645 DEGs in the GSE95728 dataset. Volcano maps were used for visualization (**Figure S3B**). Additionally, 128 DEGs were detected in the GSE34526 dataset (**Figure S3C**). Ultimately, 85 overlapping DEGs were screened (**Figure S3D**).

**Figure 6:**
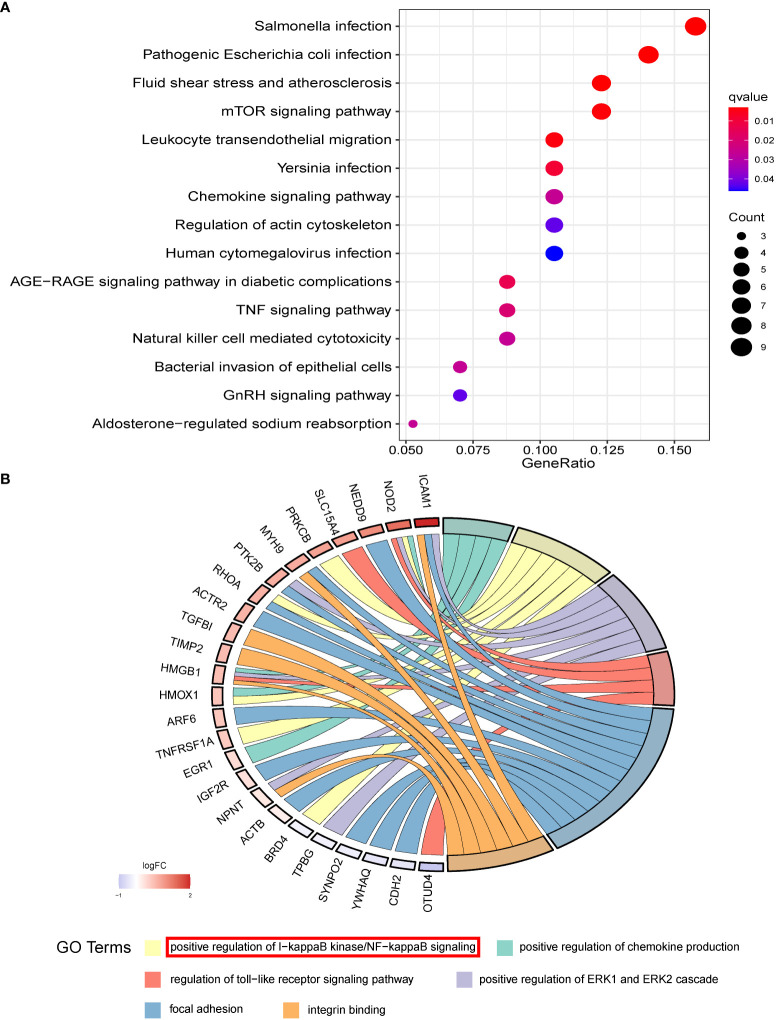
The functional enrichment results of overlapped DEGs. **(A)** The KEGG and **(B)** GO analysis of overlapped DEGs which may be potential targets of miR-93-5p.

To explore the underlying functional mechanism of miR-93-5p-related genes, we conducted KEGG and GO functional enrichment analysis. Interestingly, the KEGG analysis results indicated that DEGs were enriched in the mTOR signaling pathway, TNF signaling pathway, chemokine signaling pathway, GnRH signaling pathway, NOD-like receptor signaling pathway, PI3K-Akt signaling pathway, etc. (**Figure 6A**). Additionally, the results of GO analysis showed that the DEGs were related to the NF-κB, MAPK, and JNK signaling pathway, DNA metabolic process, ERK1 and ERK2 cascade, focal adhesion, and cell-substrate junction (**Figure 6B**).

### miR-93-5p negatively modulated the NF-κB signaling pathway

**Figure 7:**
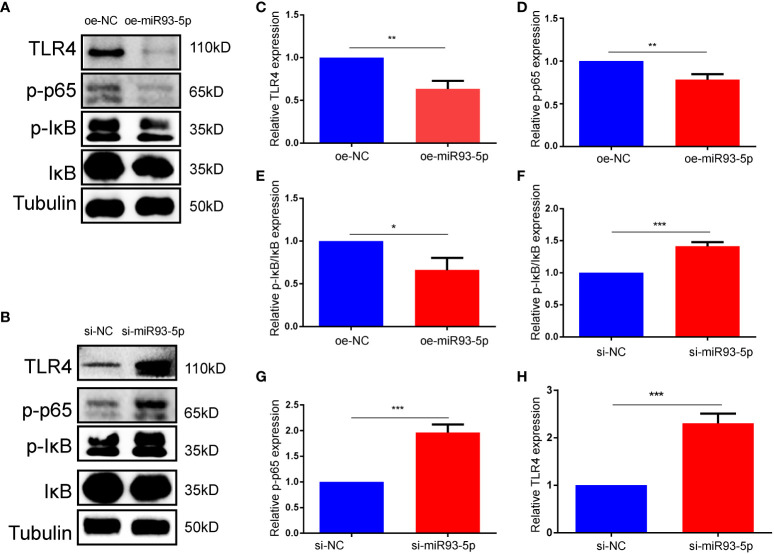
miR-93-5p negatively regulates the NF-κB signaling pathway. **(A, B)** WB results showed that the protein expression of TLR4, p-p65 and p-IκB/IκB was decreased in the oe-miR93-5p group compared with the oe-NC group, while was increased in the si-miR93-5p group compared with the si-NC. The bar charts are used to visualize the expression of **(C, F)** TLR4, **(D, G)** p-p65, **(E, H)** p-IκB/IκB. *p* < 0.05 (“*”), *p* < 0.01 (“**”), *p* < 0.001 (“***”).

Numerous studies have shown that NF-κB may induce the antiapoptotic pathways and is involved in cell death (36, 37). Additionally, articles on NF-κB and ferroptosis have identified potential hotspots and the study of NF-κB in ferroptosis may be the next topics (38). Combined with biological analysis and a literature search, we hypothesized that miR-93-5p may mediate proliferation, apoptosis and ferroptosis by acting on the NF-κB signaling pathway. Western blotting was applied to explore the association between miR-93-5p and the NF-κB signaling pathway, and the results suggested that elevated miR-93-5p inhibited TLR4, p-p65, and p-IκB/IκB levels, while silencing miR-93-5p had the opposite effect (**Figure 7**).

### NF-κB inhibitor reverses the function of miR-93-5p

**Figure 8:**
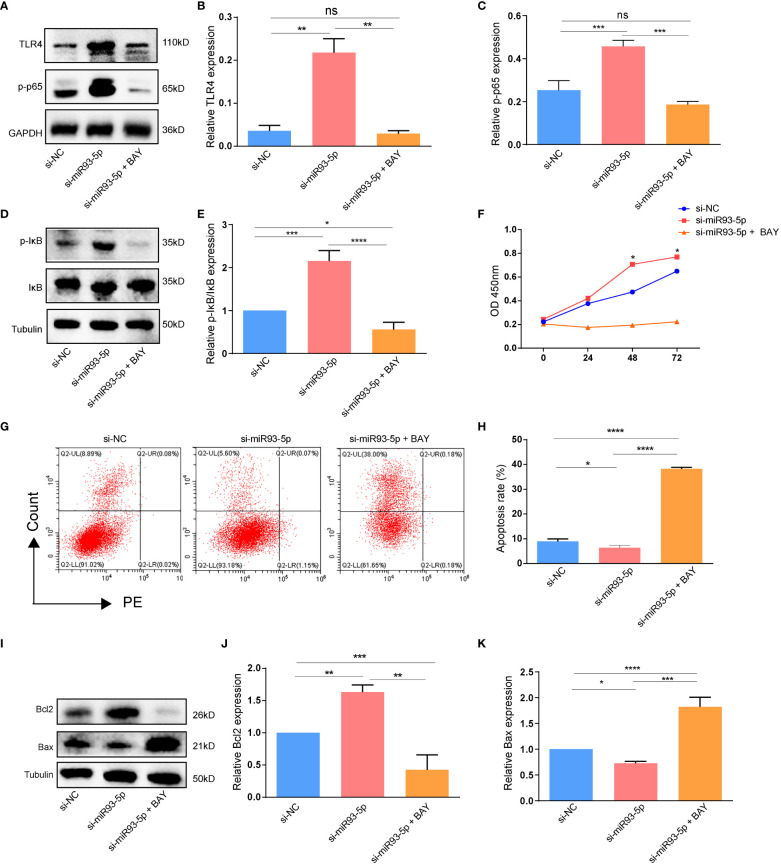
The effect of the NF-κB signaling pathway on the apoptosis. **(A–E)** WB demonstrated that the expression of TLR4, p-p65 and p-IκB/IκB were significantly inhibited by NF-κB inhibitor (BAY 11-7082). **(F)** The cell viability among three groups. **(G, H)** Flow cytometry assay showed that there was an increased apoptosis level in the si-miR93-5p + BAY group. **(I–K)** The protein expression of Bax and Bcl-2 among three groups. *p* < 0.05 (“*”), *p* < 0.01 (“**”), *p* < 0.001 (“***”), *p* < 0.0001 (“****”).

**Figure 9:**
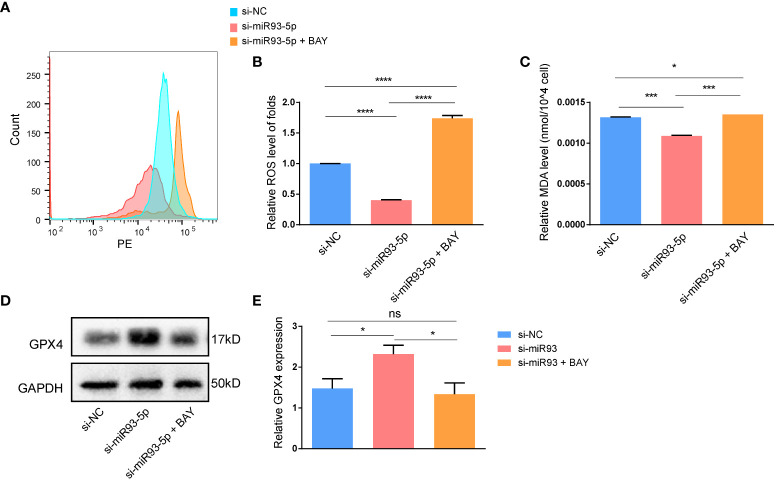
The effect of the NF-κB signaling pathway on the ferroptosis in GCs. **(A, B)** The ROS level was significantly increased in the si-miR93-5p + BAY group. The relative expression of **(C)** MDA and **(D, E)** GPX4 among three groups. *p* < 0.05 (“*”), *p* < 0.001 (“***”), *p* < 0.0001 (“****”). ns means no statistical difference.

We explored whether an NF-κB inhibitor (BAY 11-7082) could reverse the effects of miR-93-5p on GCs. WB demonstrated that compared with the si-miR93-5p group, the NF-κB signaling pathway was significantly inhibited by BAY 11-7082 (**Figures 8A–E**). As expected, when NF-κB was inhibited, apoptosis and proliferation of GCs increased and decreased, respectively (**Figures 9F–K**). Moreover, changes in MDA, ROS and GPX4 suggested that inhibition of NF-κB could reverse the effect of miR-93-5p on ferroptosis (**Figure 9**).

## Discussion

PCOS is a common reproductive endocrine metabolic disease that affects approximately 4~20% of reproductive-aged women (39). Furthermore, PCOS patients are prone to metabolic (obesity, type 2 diabetes, cardiovascular disease), reproductive (anovulation, subfertility) and psychological (depression, eating disorders) sequelae (40–44). However, to date, no universal treatment for PCOS has been developed, and no drugs are approved specifically for PCOS. Thus, exploration of the pathogenesis of PCOS could contribute to the treatment.

An increasing number of studies have focused on the role of microRNAs in PCOS. Sathyapalan et al. conducted a case-controlled study and found that the expression of miR-93-5p was elevated in the plasma of PCOS patients (31). Additionally, the expression of miR-93-5p was upregulated in GCs, follicular fluid, and adipose tissue of PCOS patients, and these changes were closely related to hyperandrogenic and insulin resistance (11, 13, 45). The latest systematic review and meta-analysis of the role of miRNAs in PCOS revealed that miR-93-5p was upregulated in PCOS without heterogeneity (12). Aberrant expression of miR-93-5p may provide new insight into exploring the mechanisms of the pathogenesis of PCOS. However, most of the analysis in the above studies were performed at the clinical level, and specific in-depth mechanistic studies have not been performed. In this study, we investigated the potential functions and mechanisms of miR-93-5p in PCOS.

GCs are the major component of the ovary, and the proliferation, development, and death of GCs play crucial roles in regulating ovarian functions. KGN cells have similar physiological characteristics to ovarian GCs and are normally used to study the function and regulatory mechanism of GCs. Apoptosis and ferroptosis are two different forms of cell death. Apoptosis is a noninflammatory programmed form of death, and it is also one of the most studied topics among cell biologists (46). It can be triggered by two distinct pathways, namely, the intrinsic (also called Bcl-2-regulated) pathway and the death receptor pathways, and these pathways ultimately in morphological and biochemical cellular alterations, which are characteristics of apoptosis (47). Apoptosis is characterized by changes in the caspase family and pro-apoptotic and anti-apoptotic members (46). In our article, we only studied the pro-apoptotic (BAX) and anti-apoptotic proteins (BCL2), and did not further explore the changes in caspase family members, which represents one of the shortcomings of the study. Compared with apoptosis, ferroptosis is a ROS-dependent form of cell death associated with lipid peroxidation and is initiated through two major pathways: the extrinsic or transporter-dependent pathway involving System XC-, and the intrinsic or enzyme-regulated pathway (19, 48). A previous study showed that the expression of GPX4 is downregulated in the uterus of pregnant patients by maternal exposure to 5α-dihydrotestosterone (49). In this study, we showed that the overexpression of miR-93-5p can promote apoptosis by reducing the expression of Bcl2 and increasing ferroptosis by downregulating GPX4, SLC7A11 and Nrf2 expression in the KGN cell line, moreover, we found that inhibition of miR-93-5p produced the reverse effect.

**Figure 10:**
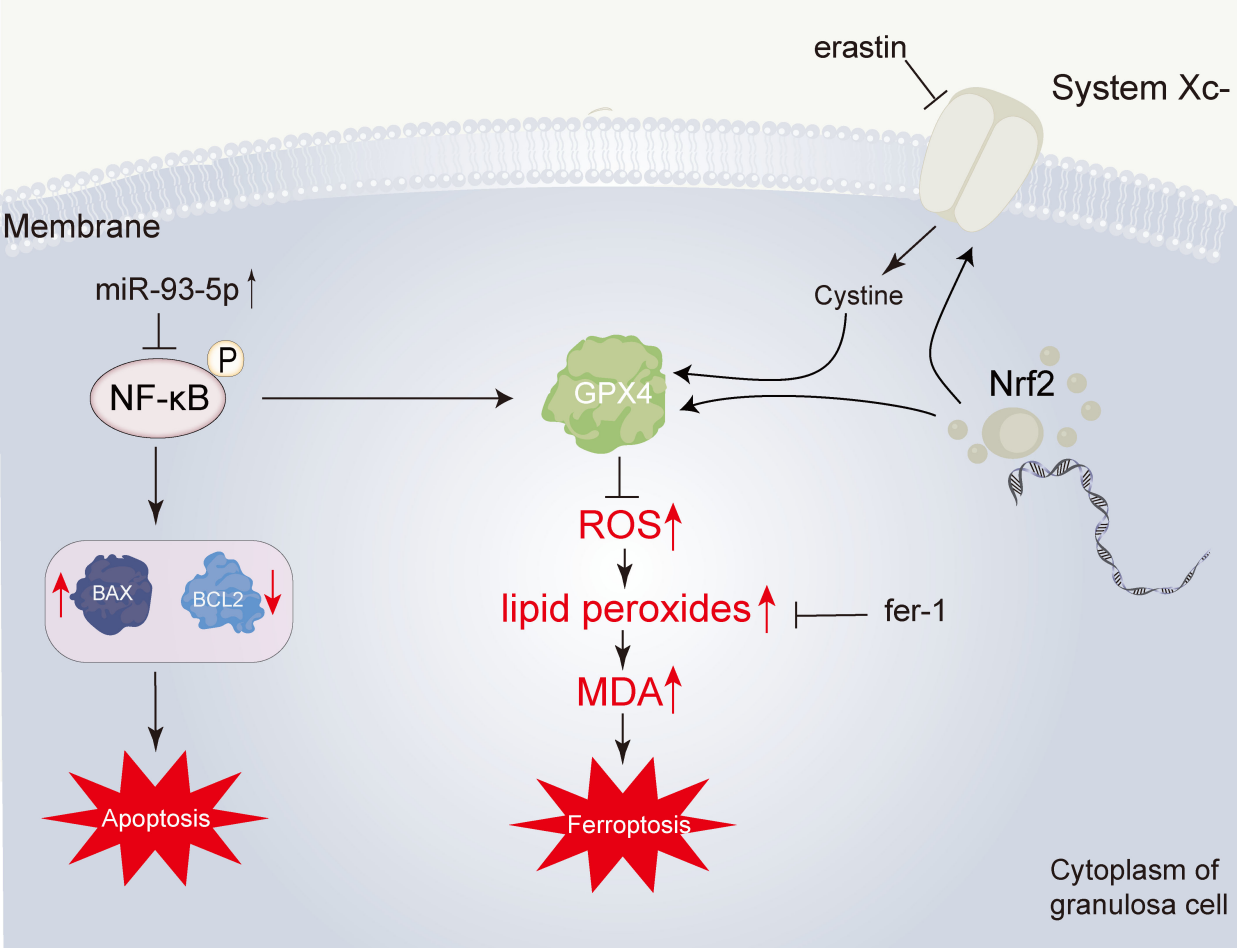
Overview of the effect of miR-93-5p in GC. The miR-93-5p is elevated in PCOS. The elevated miR-93-5p negatively regulates the NF-κB signaling pathway, inducing the increase of BAX and the decrease of BCL2, and finally leading the cell apoptosis. Additionally, when the NF-κB signaling pathway is inhibited, the transcription of GPX4 is declined. Then, the level of lipid peroxides is promoted. This process can be inhibited by ferrostatin-1 (fer-1). Lipid peroxides can be degraded into reactive aldehydes, such as malondialdehyde (MDA).

Regarding the possible mechanism involved in the role of miR-93-5p, we screened the overlapping genes between the PCOS-associated and miR-93-5p potential targets. Next, the overlapping DEGs were identified in GSE95728 and GSE34526. KEGG and GO functional enrichment analysis were also conducted. Interestingly, we noticed that the overlapping DEGs were related to the NF-κB signaling pathway and there is numerous evidence showed that the NF-κB signaling pathway is closely connected with cell apoptosis and ferroptosis (36, 50–53). Subsequently, western blotting was performed to detect whether the NF-κB signaling pathway was changed in the oe-miR93-5p and si-miR93-5p groups. The results revealed that elevated expression of the NF-κB signaling pathway in the si-miR93-5p group and decreased expression in the oe-miR93-5p group. Additionally, we proved that the NF-κB inhibitor BAY 11-7082 could reverse the effects of miR-93-5p on GCs. Collectively, our study elucidated that miR-93-5p is involved in the apoptosis and ferroptosis of GCs. Mechanistically, miR-93-5p inhibits GC proliferation and promots apoptosis and ferroptosis *via* inhibition of the NF-κB signaling pathway (**Figure 10**). miR-93-5p may be a new molecular target for improving the function of GCs in PCOS. In conclusion, our study provides a new perspective on PCOS pathogenesis and GC dysfunction.

## Data availability statement

The original contributions presented in the study are included in the article/**Supplementary Material**. Further inquiries can be directed to the corresponding author.

## Author contributions

FD and WT collected and initially screened the data. XZ, FD and YC guided the research ideas of the full text. WT and RG performed a visual analysis of the data and was the main contributor to the manuscript. ZD and DY improved the writing style and addressed grammatical errors. All authors contributed to the article and approved the submitted version.

## Funding

This research was funded by the National Natural Science Foundation of China, grant number (82071655,81860276); Key Research and Development Program of Hubei Province (2020BCB023); the Guangdong Basic and Applied Basic Research Foundation (2020B1515020001); the GDPH supporting fund (KY012021439); the 5010 grants of Sun Yat-sen University (SYSU2019003); Young Teacher Qualification Project of the Fundamental Research Funds for the Central Universities (2042020kf0088).

## Acknowledgments

We sincerely thank the data provided by the GEO, and thank the Central Laboratory of Wuhan University People’s Hospital for their help during the study.

## Conflict of interest

The authors declare that the research was conducted in the absence of any commercial or financial relationships that could be construed as a potential conflict of interest.

## Publisher’s note

All claims expressed in this article are solely those of the authors and do not necessarily represent those of their affiliated organizations, or those of the publisher, the editors and the reviewers. Any product that may be evaluated in this article, or claim that may be made by its manufacturer, is not guaranteed or endorsed by the publisher.
