# Supplementary figures and images for: MiR-93-5p promotes granulosa cell apoptosis and ferroptosis by the NF-kB signaling pathway in polycystic ovary syndrome
Source: Front Immunol. 2022 Oct 19;13:967151. doi: 10.3389/fimmu.2022.967151 (PMC9626535; doi:10.3389/fimmu.2022.967151)

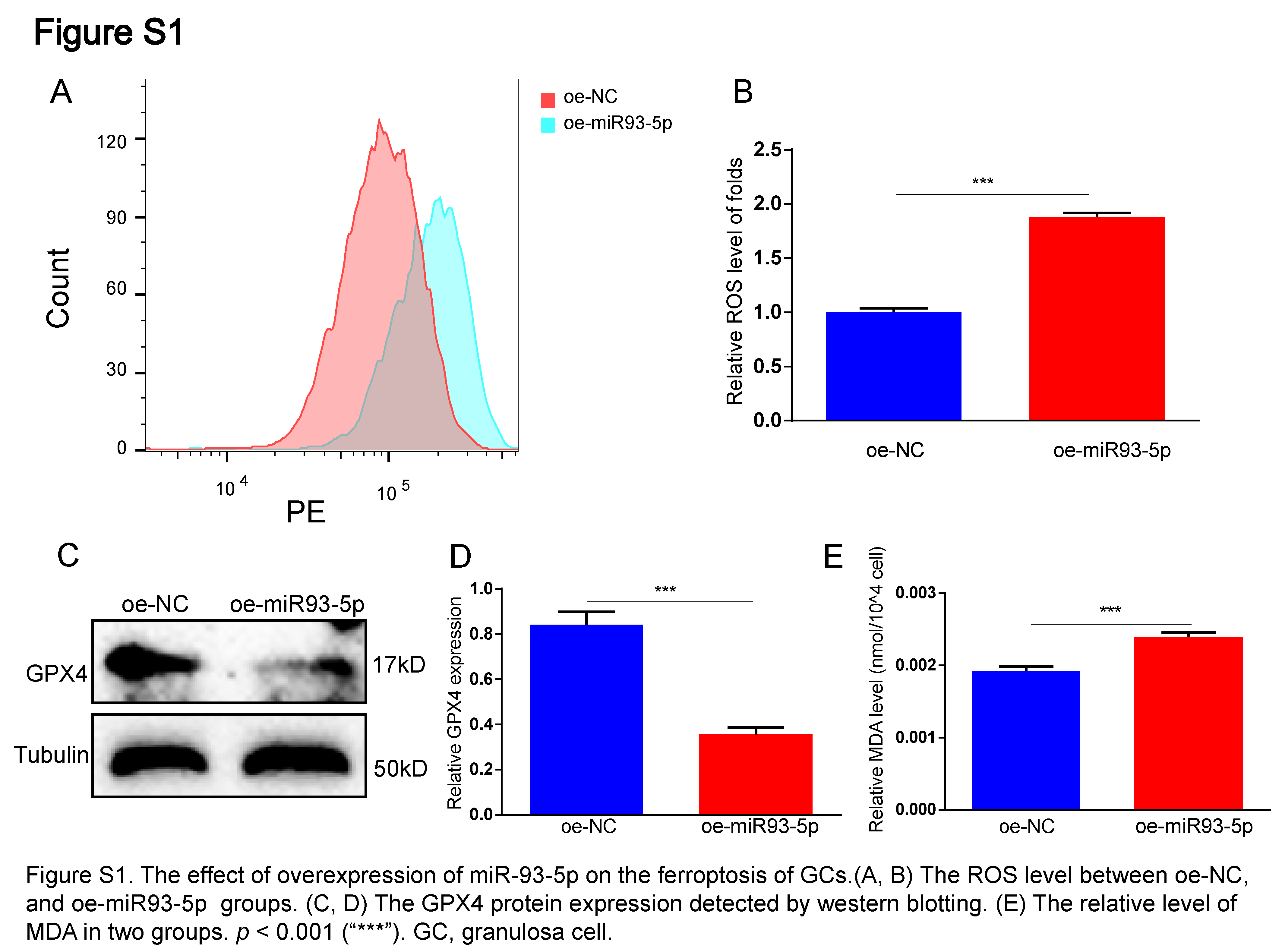

Supplement: Supplementary file 1 [file Image_1.tif]

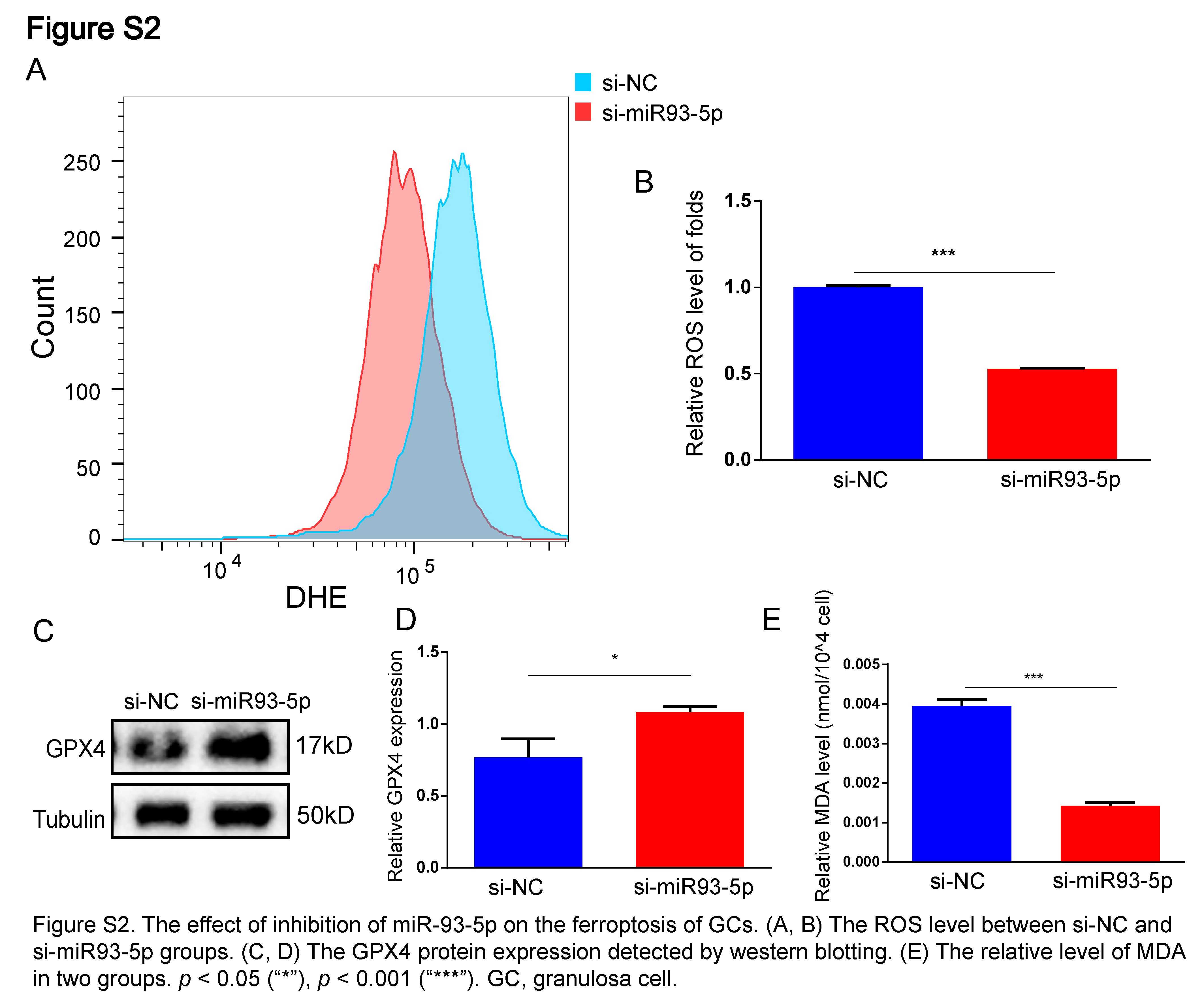

Supplement: Supplementary file 2 [file Image_2.tif]

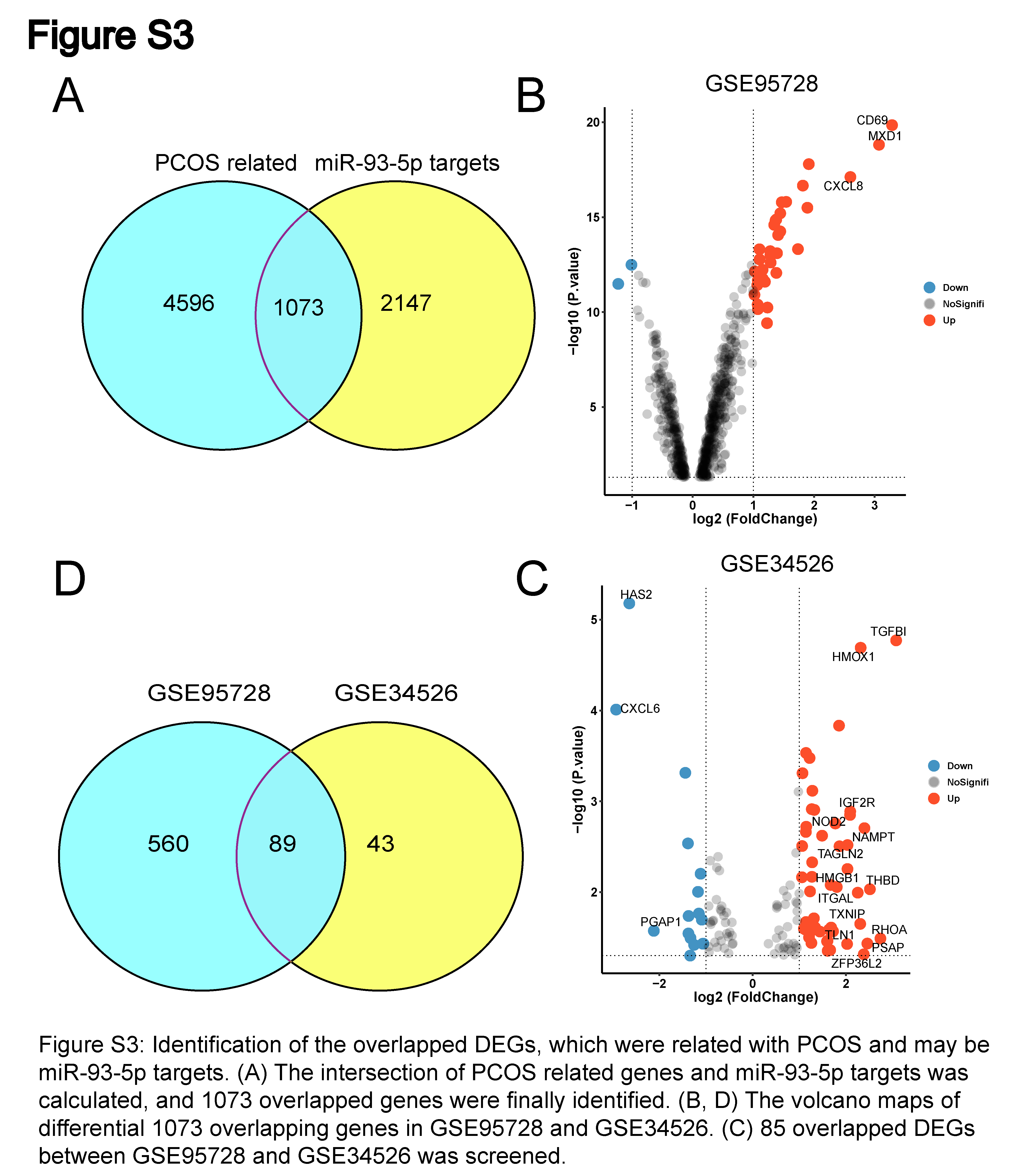

Supplement: Supplementary file 3 [file Image_3.tif]
